# The relationship between serum uric acid and cognitive function in patients with chronic heart failure

**DOI:** 10.1186/s12872-020-01666-z

**Published:** 2020-08-20

**Authors:** Weihua Niu, Huifeng Yang, Chengzhi Lu

**Affiliations:** 1 First Center Clinic College of Tianjin Medical University, Tianjin First Central Hospital, No 24 Fukang Road, Nankai District, Tianjin, 300192 China; 2 Department of Cardiology, Tianjin First Central Hospital, No 24 Fukang Road, Nankai District, Tianjin, 300192 China; 3 School of Nursing, Tianjin University of Traditional Chinese Medicine, No 10 Panyang Road, West District, Tuanbo New Town, Jinghai District, Tianjin, 301617 China

**Keywords:** Cognitive impairment, Heart failure, Serum uric acid

## Abstract

**Background:**

Evidence has shown that serum uric acid (UA) is associated with cognitive function, but this finding remains debatable. Serum UA is commonly elevated in patients with chronic heart failure (CHF), especially in men. However, the relationship between serum UA and cognitive function in CHF populations and stratified by sex are unclear. We aimed to examine whether serum UA was independently associated with cognitive function in CHF populations after controlling for demographic, medical and psychological variables and whether there was a sex difference in the association between serum UA and cognitive function among male and female CHF patients.

**Methods:**

One hundred ninety-two hospitalized patients with CHF underwent an assessment of cognitive function using the Montreal Cognitive Assessment (MoCA) and the determination of serum UA. Hyperuricemia was defined as serum UA ≥7 mg/dl in men and ≥ 6 mg/dl in women. Multiple linear hierarchical regression analyses were conducted to examine the independent association between serum UA and cognitive function in CHF populations and stratified by sex.

**Results:**

The mean serum UA concentration of participants was 7.3 ± 2.6 mg/dL. The prevalence of hyperuricemia was 54.7% (105 of 192) in CHF patients, 52.9% (64 of 121) in men, and 57.7% (41 of 71) in women. In the total sample, higher serum UA was associated with poorer cognitive function independent of demographic, medical and psychological variables (β = − 0.130, ΔR^2^ = 0.014, *p =* 0.015). In sex-stratified groups, elevated serum UA was independently associated with worse cognitive function in men (β = − 0.247, ΔR^2^ = 0.049, *p =* 0.001) but not in women (β = − 0.005, ΔR^2^ = 0.000, *p =* 0.955).

**Conclusions:**

Higher serum UA is independently associated with poorer cognitive function in CHF populations after adjusting for confounding variables. Furthermore, elevated serum UA is independently related to worse performance on cognitive function in men but not in women. More longitudinal studies are needed to examine the association between serum UA and cognitive function in CHF populations and stratified by sex.

## Background

Chronic heart failure (CHF) is a significant health problem that affects an estimated 6.5 million Americans aged 20 years or older [1]. CHF is associated with frequent hospital admissions, reduced quality of life, and huge economic burden to the healthcare system [2]. There is increasing evidence that CHF is also related to cognitive impairment, dementia, and Alzheimer’s disease [3, 4]. Conversely, cognitive impairment is associated with adverse outcomes in CHF patients, including poor self-care [5], poor medication [6] and treatment adherence [7], and high mortality [8].

Previous work has demonstrated that many medical comorbidities play an important role in the pathogenesis of cognitive impairment in CHF patients, including hypertension [9], type 2 diabetes mellitus [10], atrial fibrillation [11], obesity [12], chronic renal dysfunction [13], anemia [14], hyponatremia [15], depression [16], and sleep apnea [17]. However, the underlying mechanisms remain unclear.

Uric acid (UA) is the final product of purine catabolism and is excreted by the kidneys. In recent years, evidence has consistently shown that the level of serum UA is associated with cognitive function, but this relationship remains controversial. Some studies have indicated that the higher level of serum UA is associated with better cognitive performance owing to the antioxidant property of serum UA [18–23]. Specifically, a retrospective study including 1374 elderly showed that higher levels of serum UA were related to better cognitive function after controlling for cardiovascular risk factors [18]. A large cross-sectional study consisting of 10,039 Chinese community-dwelling participants aged ≥55 years indicated that high serum UA was associated with a decreased risk of cognitive impairment after adjusting for age, sex, lifestyle, relevant diseases and the apolipoprotein E (APOE) ε4 allele [19]. A prospective cohort study found that elevated serum UA predicted a decreased risk of dementia after adjusting for several cardiovascular risk factors among 4618 participants aged ≥55 years and that higher serum UA at baseline predicted better cognitive performance later in life in the absence of cardiovascular risk factors among 1724 participants who remained free of dementia during follow-up [21]. Moreover, a case-control study demonstrated that serum UA levels were significantly lower in Alzheimer’s disease (AD) patients compared to healthy controls [22]. In addition, a nested case-control study including 388 new Parkinson’s disease (PD) patients (202 men and 186 women) and 1267 controls showed that higher serum UA predicted a lower risk of developing PD in men but not in women [23]. On the contrary, some studies have demonstrated that elevated serum UA is related to poorer cognitive function by the pro-oxidant character of serum UA [24–27]. Specifically, a cross-sectional study of 1144 participants aged ≥70 years indicated that higher serum UA was associated with worse cognitive function among women but not among men, especially among women with cardiovascular disease [24]. Another study including 288 healthy young elderly from the historical cohort of the Brisighella Heart Study found a tight inverse relationship between circulating serum UA levels and cognitive performance [25]. Moreover, a population-based cohort study of 1598 healthy older people from the community demonstrated a positive association between serum UA and the risk of dementia, especially vascular or mixed dementia over 12 years of follow-up [27].

Elevated serum UA or hyperuricemia has been well demonstrated to be common in CHF patients [28, 29] and a marker of adverse outcomes in CHF patients, including rehospitalization, higher all-cause mortality, and higher cardiovascular mortality [29–31]. However, the specific role of serum UA in cognitive function among CHF populations has not been addressed. Enlightening the potential beneficial or harmful effects of serum UA on cognitive function in CHF patients may provide a better understanding of potential pathogenesis and interventions for cognitive impairment.

Moreover, previous work has indicated that serum UA may have different effect on cognitive function in men and women [20, 24, 32]. One study demonstrated that there was an inverse correlation between serum UA and the risk of cognitive impairment only in male participants [20]. Another study found that serum UA was potentially beneficial for attention/processing speed only among older men [32]. However, a cross-sectional study indicated that higher serum UA predicted worse cognitive performance in women [24]. Whereas, a sex-stratified analysis on the relationship between serum UA and cognitive function in CHF patients has been not explored. Such analysis is important because elevated serum UA is more common [29, 33] and associated with poorer ejection fraction [34] in male CHF patients.

Therefore, the present study aimed to examine the independent association between serum UA and cognitive function in a sample of hospitalized patients with CHF and stratified by sex.

## Methods

### Participants

This study was a descriptive cross-sectional design. Between August 2019 and December 2019, a convenience sample of 192 patients hospitalized for symptomatic HF with reduced ejection fraction (HFrEF) or with preserved ejection fraction (HFpEF) and confirmed by the cardiologists was selected from the cardiology inpatient department of Tianjin First Central Hospital, a large (more than 1500 beds) and comprehensive (47 clinical professional departments) hospital in Tianjin, China.

The inclusion criteria were as follows: (1) aged 45 to 85 years at enrollment, (2) a primary diagnosis of HF as defined by the clinical criteria of Chinese Heart Failure Diagnosis and Treatment Guidelines (typical symptoms (e.g., dyspnea, fatigue, or decreased exercise capacity), signs (e.g., edema or rale), and chest X-ray evidence of congestion or structural and functional abnormalities on cardiac ultrasound) [35], (3) New York Heart Association (NYHA) functional class II–IV of ≥3 months duration. The age range of 45–85 years was used to maximize the number of participants with some cognitive impairment and to minimize the number of participants with dementia [36].

The exclusion criteria were as follows: (1) history of cardiac surgery within past 3 months, (2) significant neurological disorder (e.g. Alzheimer’s disease, dementia, stroke, seizures, multiple sclerosis), (3) head injury with > 10 min loss of consciousness, (4) severe psychiatric disorder (e.g. schizophrenia, bipolar disorder), (5) past or current substance abuse/dependence, (6) renal failure (estimated glomerular filtration rate < 15 ml/min/1.73 m^2^), (7) past or current uric acid medication (e.g. allopurinol, benzbromarone, febuxostat), (8) inability to answer questionnaires independently due to language barriers, or visual/hearing acuity.

### Measures

#### Cognitive function

The Beijing version of the Montreal Cognitive Assessment (MoCA) was used to assess cognitive function. This tool has been shown to be sensitive for screening cognitive impairment in patients with HF [37]. In the instrument development study, the MoCA had high sensitivity (90%) and specificity (78%) for detecting mild cognitive impairment [38]. The study undertaken in Eastern China indicated that The Beijing version of MoCA had a sensitivity of 92% and a specificity of 85% for identifying mild cognitive impairment [39]. The MoCA examines eight cognitive domains, including visuospatial/executive function, naming, memory, attention, language, abstraction, delayed recall, and orientation. Total possible score ranges form 0 to 30 points, with a score below 26 indicating cognitive impairment [37, 38]. A low educational level is corrected by adding 1 point to the total score for ≤12 years of formal education [38].

#### Serum uric acid and other biochemistry indicators

Blood samples were collected after an overnight fasting (≥8 h) using standardized equipment and procedures for measuring serum UA and other biochemistry indicators (serum hemoglobin, hypersensitive C-reactive protein and creatinine). All biochemistry analyses were performed in the central biochemistry laboratory of Tianjin First Central Hospital using standard automated procedures. We defined as hyperuricemia as serum UA ≥7 mg/dl (420 μmol/L) in men and ≥ 6 mg/dl (360 μmol/L) in women [40]. The estimated glomerular filtration rate (eGFR) was calculated using the Modification of Diet in Renal Disease Study equation [41].

#### Depressive symptoms

The Chinese version of the Zung Self-Rating Depression Scale (SDS) was used to measure depressive symptoms. The SDS consisted of 20 items with each item rated on a four-point Likert scale ranging from1 to 4. The total raw score ranges from 20 to 80, and the standard score is the integer portion of the product of 1.25 and the total raw score. According to the Chinese norm, depression is defined as the standard score ≥ 53 [42].

#### Demographic and medical characteristics

Demographic and medical variables were collected through participant self-report, and supplemented and corroborated by medical record review. Through these methods, demographic (e.g. age, gender) and medical history (e.g. hypertension, diabetes, atrial fibrillation) were ascertained. The severity of HF was assessed with NYHA functional class and left ventricular ejection fraction (LVEF). Body mass index (BMI) was calculated as the ratio of weight (in kilograms) to height squared (in square meters).

### Procedures

The study was in accordance with the declaration of Helsinki, and informed consent was obtained from all patients before enrolment. All study procedures were approved by The Medical Ethics Committee of Tianjin First Central Hospital. In additional to medical record review, participants completed demographic, medical and psychological self-report measures. The psychometric evaluation of the MoCA was then administered to assess visuospatial/executive function, naming, memory, attention, language, abstraction, delayed recall, and orientation. All measures were conducted in a clinical examination room within 24 h before discharge from the hospital. In order to improve the quality of reporting in observational studies, the present study was organized in a manner compliant with the Strengthening the Reporting of Observational Studies in Epidemiology (STROBE) statement.

### Statistical analyses

The SPSS version 17.0 (SPSS Inc., Chicago, Illinois, USA) was used to perform statistical analyses. Descriptive statistics were used to illustrate the characteristics of the study participants. Normally distributed continuous variables were shown with mean ± standard deviation (SD), and non-normally continuous data were described with medians and quartiles. Categorical variables were shown with frequencies and percentages.

Independent sample *t*-test, Mann-Whitney U test and Pearson’s chi-square test or Fisher’s exact test were used to analyze the differences of the study variables between male and female CHF patients.

To explore the independent association of serum UA on cognitive function in CHF patients, a multiple linear hierarchical regression analysis was preferable to be conducted [43]. Specifically, the MoCA score was included as the dependent variable, while serum UA was included as independent variable. To control for potential confounders of cognitive function, age, gender, educational level, NYHA function class, LVEF, BMI, serum hemoglobin, hypersensitive C-reactive protein (hs-CRP), eGFR, the diagnostic history of hypertension, type 2 diabetes mellitus, and atrial fibrillation, and SDS score were included as covariates.

Following this analysis, two separate multiple linear hierarchical regression analyses were performed to determine the independent effect of serum UA on cognitive function in male and female CHF patients. Each model was examined by entering potential confounders (age, educational level, NYHA function class, LVEF, BMI, serum hemoglobin, hs-CRP, eGFR, the diagnostic history of hypertension, type 2 diabetes mellitus, and atrial fibrillation, SDS score) into block 1 and entering an independent variable (serum UA) into block 2. Change in R^2^ from block 1 to block 2 determined the significance of the incremental predictive validity of serum UA over the covariates.

Sample size is calculated based on the number of independent variables included in the regression equation whereby the number of participants should be at least 5–10 times of the number of independent variables entered into the regression equation [44]. The numbers of independent variables were 14 in the first regression model, and 13 in the second and third regression model, thus, sample size was sufficient for the first regression model including 192 CHF patients, for the second regression model including 121 men, and for the third regression model including 71 women.

Since hs-CRP levels were not normally distributed, we used a log10 transformation for their use in the multiple linear hierarchical regression analyses. The results of multiple linear hierarchical regression analyses were presented by beta coefficients (β) and 95% confidence intervals (CI) for β. A *p*-value of < 0.05 (two-sided) was considered to be statistically significant.

## Results

### Demographic, medical, and psychological differences between male and female CHF patients

A total of 192 CHF patients (mean age 66.1 ± 10.6 years, 63.0% male) were recruited for the study. As presented in Table 1, the current sample had a mean serum UA concentration of 7.3 ± 2.6 mg/dL. Hyperuricemia was present in 54.7% (105 of 192) of CHF patients, 52.9% (64 of 121) of men, and 57.7% (41 of 71) of women.

**Table 1:** Characteristics of participants (*n* = 192)

	Total sample (*n* = 192)	Men (*n* = 121)	Women (*n* = 71)	*P*-value
**Demographic characteristics**				
Age (years)	66.1 ± 10.6	64.1 ± 10.5	69.5 ± 9.9	0.001^a^
Male	121 (63.0)	–	–	
Educational level (years)				0.000^b^
0–6	53 (27.6)	18 (14.9)	35 (49.3)	
7–9	53 (27.6)	37 (30.6)	16 (22.5)	
10–12	65 (33.9)	49 (40.5)	16 (22.5)	
≥ 13	21 (10.9)	17 (14.0)	4 (5.6)	
**Medical and psychological characteristics**				
NYHA functional class				0.212^b^
II	35 (18.2)	24 (19.8)	11 (15.5)	
III	106 (55.2)	70 (57.9)	36 (50.7)	
IV	51 (26.6)	27 (22.3)	24 (33.8)	
LVEF (%)	37.6 ± 12.3	36.0 ± 11.8	40.4 ± 12.7	0.017^a^
BMI (Kg/m^2^)	25.0 ± 4.5	25.2 ± 4.1	24.7 ± 5.1	0.540^c^
Hemoglobin(g/dL)	13.0 ± 2.4	13.8 ± 2.3	11.7 ± 2.1	0.000^a^
hs-CRP (mg/L)	4.4 (1.8–16.4)	4.2 (1.4–12.4)	5.2 (2.4–19.3)	0.056^d^
eGFR (ml/min/1.73m^2^)	72.8 ± 30.8	74.1 ± 31.6	70.7 ± 29.4	0.457^a^
Serum UA (mg/dl)	7.3 ± 2.6	7.6 ± 2.6	6.8 ± 2.5	0.044^a^
Hyperuricemia	105 (54.7)	64 (52.9)	41 (57.7)	0.514^b^
Hypertension	128 (66.7)	79 (65.3)	49 (69.0)	0.597^b^
Type 2 diabetes mellitus	84 (43.8)	57 (47.1)	27 (38.0)	0.221^b^
Atrial fibrillation	73 (38.0)	47 (38.8)	26 (36.6)	0.759^b^
SDS score	54.7 ± 10.0	53.9 ± 10.1	56.1 ± 9.8	0.147^a^
**Cognitive characteristics**				
MoCA score	25.1 ± 2.9	25.8 ± 2.7	23.9 ± 2.9	0.000^a^
Cognitive impairment	103 (53.6)	51 (42.1)	52 (73.2)	0.000^b^

Continuous variables represented as mean ± SD and median (Q1–Q3), categorical variables as n(%)

*NYHA* New York Heart Association, *LVEF* left ventricular fraction, *BMI* body mass index, *hs-CRP* hypersensitive C-reactive protein, *eGFR* estimated glomerular filtration rate, *UA* uric acid, *SDS* Self-Rating Depression Scale, *MoCA* Montreal Cognitive Assessment

^a^*t-*test

^b^Pearson’s chi-square test

^c^*t*’-test

^d^Mann–Whitney U-test

No significant differences were determined between male and female CHF patients in terms of NYHA functional class, BMI, hs-CRP, eGFR, hyperuricemia, hypertension, type 2 diabetes mellitus, atrial fibrillation, or SDS score. However, male CHF patients were younger (*p* = 0.005), and had different educational level (*p* = 0.000), lower LVEF level (*p* = 0.017), higher hemoglobin level (*p* = 0.000), and higher serum UA level (*p* = 0.044).

### Cognitive function

In term of cognitive function, the mean MoCA score of the total participants was 25.1 ± 2.9. Based on the MoCA score, 53.6% (103 of 192) of the whole study population had cognitive impairment, including 42.1% (51 of 121) of men and 72.3% (52 of 71) of women. Specifically, women had lower MoCA score (*p* = 0.000), and were more likely to have cognitive impairment compared with men (*p* = 0.000) (Table 1).

### Serum UA is independently associated with cognitive function in CHF patients

A multiple linear hierarchical regression analysis was conducted to determine the predictive validity of serum UA on cognitive function in CHF patients. As presented in Table 2, block 1 with demographic, medical and psychological variables was significantly associated with cognitive function (R^2^ = 0.584, *p* = 0.000). Specifically, older age (β = − 0.353, *p =* 0.000), lower educational level (β = 0.297, *p =* 0.000), higher hs-CRP (β = − 0.188, *p =* 0.000), and higher SDS score (β = − 0.261, *p =* 0.000) emerged as significant predictors of poorer cognitive function in CHF patients.

**Table 2:** A hierarchical regression of serum UA predicting cognitive function in patients with CHF (*n* = 192)

	Cognitive function					
	β	95% CI		R^**2**^	ΔR^**2**^	*P*-value
		Lower	Upper			
**Block 1**	**–**	**–**	**–**	**0.584**	**–**	0.000**
Age	−0.353	− 0.474	− 0.231			0.000**
Gender	−0.101	−0.213	0.012			0.080
Educational level	0.297	0.188	0.406			0.000**
NYHA function class	−0.035	−0.145	0.076			0.536
LVEF	0.013	−0.100	0.125			0.825
BMI	0.070	−0.041	0.180			0.214
Hemoglobin	−0.084	−0.208	0.040			0.184
hs-CRP	−0.188	−0.292	− 0.084			0.000**
eGFR	−0.002	−0.111	0.107			0.967
Hypertension	−0.073	−0.177	0.032			0.171
Type 2 diabetes mellitus	−0.009	−0.108	0.090			0.852
Atrial fibrillation	−0.080	−0.190	0.030			0.154
SDS score	−0.261	−0.379	− 0.142			0.000**
**Block 2**	**–**	**–**	**–**	**0.598**	**0.014**	0.015*
Serum UA	−0.130	−0.235	− 0.025			0.015

*UA* uric acid, *CHF* chronic heart failure, *NYHA* New York Heart Association, *LVEF* left ventricular fraction, *BMI* body mass index, *hs-CRP* hypersensitive C-reactive protein, *eGFR* estimated glomerular filtration rate, *SDS* self-rating depression scale

*β* standardized coefficients, *CI* confidence interval

* *P* < 0.05; ** *P* < 0.01

After adjusting for demographic, medical and psychological variables, serum UA showed significant predictive validity for cognitive function in CHF patients (β = − 0.130, ΔR^2^ = 0.014, *p =* 0.015).

### Serum UA is independently associated with cognitive function in male but not in female CHF patients

Two separate multiple linear hierarchical regression analyses controlling for demographic, medical and psychological characteristics were conducted to clarify the independent effect of serum UA on cognitive function in male and female CHF patients.

In male CHF patients, demographic, medical and psychological characteristics in block 1 made significant contributions to the prediction of cognitive function, explaining 51.9% of the variance (R^2^ = 0.519, *p* = 0.000). Specifically, older age (β = − 0.286, *p =* 0.001), lower educational level (β = 0.287, *p =* 0.000), atrial fibrillation (β = − 0.174, *p =* 0.029), and higher SDS score (β = − 0.293, *p =* 0.001) were associated with poorer cognitive function. After adjusting demographic, medical and psychological characteristics, serum UA significantly contributed an additional 4.9% of the variance on the prediction of cognitive function in male CHF patients (β = − 0.247, ΔR^2^ = 0.049, *p* = 0.001) (Table 3).

**Table 3:** A hierarchical regression of serum UA predicting cognitive function in male CHF patients (*n* = 121)

	Cognitive function					
	β	95% CI		R^**2**^	ΔR^**2**^	*P*-value
		Lower	Upper			
**Block 1**	**–**	**–**	**–**	**0.519**	**–**	0.000**
Age	−0.286	−0.453	− 0.119			0.001**
Educational level	0.287	0.142	0.432			0.000**
NYHA function class	−0.051	−0.201	0.099			0.501
LVEF	−0.080	−0.235	0.074			0.304
BMI	0.074	−0.081	0.229			0.344
Hemoglobin	−0.098	−0.259	0.062			0.228
hs-CRP	−0.140	−0.282	0.003			0.054
eGFR	0.028	−0.122	0.179			0.712
Hypertension	−0.046	−0.188	0.097			0.528
Type 2 diabetes mellitus	0.026	−0.112	0.165			0.710
Atrial fibrillation	−0.174	−0.329	− 0.019			0.029*
SDS score	−0.293	−0.459	− 0.126			0.001**
**Block 2**	**–**	**–**	**–**	**0.568**	**0.049**	0.001**
Serum UA	−0.247	−0.388	− 0.106			0.001**

*UA* uric acid, *CHF* chronic heart failure, *NYHA* New York Heart Association, *LVEF* left ventricular fraction, *BMI* body mass index, *hs-CRP* hypersensitive C-reactive protein, *eGFR* estimated glomerular filtration rate, *SDS* self-rating depression scale

*β* standardized coefficients, *CI* confidence interval

* *P* < 0.05; ** *P* < 0.01

In female CHF patients, several demographic, medical and psychological characteristics in block 1 was significantly related to cognitive function (R^2^ = 0.672, *p* = 0.000), including age (β = − 0.479, *p* = 0.000), educational level (β = 0.334, *p* = 0.000), hs-CRP (β = − 0.234, *p* = 0.012), and depression (β = − 0.260, *p* = 0.008). However, after controlling for demographic, medical and psychological variables, serum UA was not independently related to cognitive function (β = − 0.005, ΔR^2^ = 0.000, *p* = 0.955) (Table 4).

**Table 4:** A hierarchical regression of serum UA predicting cognitive function in female CHF patients (*n* = 71)

	Cognitive function					
	β	95% CI		R^2^	ΔR^2^	*P*-value
		Lower	Upper			
**Block 1**	**–**	**–**	**–**	**0.672**	**–**	0.000**
Age	−0.479	−0.677	− 0.282			0.000**
Educational level	0.334	0.173	0.495			0.000**
NYHA function class	−0.026	−0.209	0.157			0.774
LVEF	0.140	−0.041	0.320			0.128
BMI	0.073	−0.111	0.257			0.430
Hemoglobin	0.005	−0.184	0.193			0.961
hs-CRP	−0.234	−0.415	− 0.053			0.012*
eGFR	−0.128	−0.317	0.062			0.183
Hypertension	−0.115	−0.291	0.061			0.195
Type 2 diabetes mellitus	0.008	−0.164	0.180			0.925
Atrial fibrillation	0.033	−0.142	0.208			0.704
SDS score	−0.260	−0.451	− 0.070			0.008**
**Block 2**	**–**	**–**	**–**	**0.672**	**0.000**	**0.955**
Serum UA	−0.005	−0.185	0.175			0.955

*UA* uric acid, *CHF* chronic heart failure, *NYHA* New York Heart Association, *LVEF* left ventricular fraction, *BMI* body mass index, *hs-CRP* hypersensitive C-reactive protein, *eGFR* estimated glomerular filtration rate, *SDS* self-rating depression scale

*β* standardized coefficients, *CI* confidence interval

* *P* < 0.05; ** *P* < 0.01

## Discussion

Previous studies on the association between serum UA and cognitive function have conflicting results and no study so far has performed analyses among CHF populations. To the best of our knowledge, the current study is the first to show that elevated serum UA is independently associated with poorer performance on cognitive function in CHF patients after adjusting for demographic, medical, and psychological characteristics. Furthermore, another new finding of the present study is that elevated serum UA is independently associated with poorer performance on cognitive function in male CHF patients but not in female CHF patients.

In recent years, there is increasing evidence that serum UA may prevent the decline of cognitive function owing to its antioxidant efficacy or may worsen cognitive function by gaining pro-oxidant character. As the consequence, the association between serum UA and cognitive function remains controversial. Our study is consistent with several previous studies–mainly from elderly–showing that high serum UA is related to an increased risk of cognitive impairment. Indeed, a cross-sectional study including 1016 community-dwelling older persons demonstrated that high serum UA predicted an increased risk to suffer from a dementia syndrome [45]. Similarly, a population-based cohort study of 3720 healthy Urban persons aged 30-64y indicated that a higher baseline serum UA predicted poorer cognitive function over-time in the area of visual memory/visuo-construction [32]. Furthermore, Afsar et al. showed that serum UA was independently and inversely associated with cognitive function in patients with chronic kidney disease [46]. Additionally, a meta-analysis of 11 case-control studies found that there was no difference on the levels of serum UA between AD patients and healthy controls, but that serum UA might decrease in AD patients after an appropriate interpretation [47]. In the light of these studies, it can be said that the antioxidant properties of UA are replaced by strong pro-oxidant effects for higher circulating concentrations of serum UA [48], which may be potentially associated with structural and functional brain changes [49].

However, several studies have reported results in contrast to our current study. Some cross-sectional studies have suggested that higher uric acid levels are a protective factor for cognitive impairment in the elderly [19, 50, 51]. Moreover, serum UA levels have been found to be low in subjects with established AD or vascular cognitive impairment [52, 53]. In addition, Huang et al. found that increased serum UA was related to a decreased risk of MCI among T2DM patients whose serum UA level was below the cut-point (388.63 μmol/L) [54]. Furthermore, Some case-control studies have demonstrated that higher serum UA levels are linked to a decreased risk of cognitive impairment in the elderly [22, 55, 56]. Specifically, Al-khateeb et al. found that serum UA levels were significantly lower in AD patients compared to healthy controls [22]. A random sampling study including 58 patients with mild cognitive impairment (MCI) and 57 healthy elderly indicated that a high uric acid level was associated with a lower risk for MCI [55]. Wang et al. suggested that hyperuricemia was a protective factor for mild cognitive impairment (MCI) in non-obese elderly [56]. In addition, some prospective cohort studies have indicated that increased baseline serum UA level is associated with subsequently improved cognitive performances in patients with preexisting cardiovascular disease [57] and PD [58]. Furthermore, a meta-analysis including 21 case-control and 3 cohort studies indicated that higher serum UA level is a protective factor of AD [59]. These findings question the hypothesis that circulating serum UA levels may potentially involve in the pathophysiology of cognitive impairment, and further longitudinal studies are needed to corroborate such results.

Although the exact mechanisms accounting for above-mentioned apparent paradox are unclear, many factors play an important role in the controversial results, including sample sizes, dementia groups (such as AD, PD, and vascular dementia), age, and cardiovascular risk factors [60]. In CHF populations, several potential mechanisms may explain the inverse and independent association between serum UA and cognitive function. One potential explanation is the presence of white matter lesions because of high circulating concentrations of serum UA. This hypothesis is supported by the Rotterdam Scan Study which reported that elevated serum UA was related to white matter atrophy and worse cognition [61]. Another study also suggested that higher serum UA remained to be associated with greater white matter hyperintensities after adjusting for age, sex, race, education, hypertension, diabetes, alcohol abuse, smoking, and body mass [49]. Another possible mechanism may be the generation of inflammation induced by serum UA, as evidence shows that serum UA contributes to inflammatory markers [62] and ultimately result in cognitive dysfunction [63]. In addition, oxidative stress may be implicated, because serum UA has be shown to induce oxidative stress, which has been reported to increase the risk of endothelial dysfunction [48, 64] and result in cognitive impairment [65]. Furthermore, elevated serum UA levels are associated with an increased risk for vascular disease, such as hypertension, diabetes and chronic kidney disease [66, 67], which in turn contribute to vascular cognitive impairment [68]. Future studies are needed to explain the specific mechanisms by which serum UA contributes to cognitive impairment above and beyond HF-related pathology.

In addition, our results indicated an inverse association between serum UA and cognitive function only in male CHF patients, which is in contrast with the results form a study in which an inverse association between serum UA and cognitive function only among women, especially with cardiovascular diseases [24]. A possible explanation for the discrepancy could be the different characteristics of participants and instruments used to measure cognitive function, and further longitudinal studies are needed to corroborate such results. The exact mechanisms by which serum UA is independently associated with worse cognitive function in male CHF patients are unclear, but one potential explanation is that higher serum UA may result in lower cardiac output and ultimately result in lower cerebral blood flow in male CHF patients. Evidence shows that serum UA is negatively associated with LVEF in male CHF patients but not in female CHF patients [34]. In the present study, male CHF patients had higher serum UA than female CHF patients. Similarly, Hamaguchi et al. [29] and Vaduganathan et al. [33] also found that serum UA was commonly elevated in male CHF patients. Therefore, male CHF patients may have lower cardiac output because of higher serum UA levels, which may ultimately result in lower cerebral blood flow.

### Limitations

Our study had several limitations. First, the present study was a cross-sectional design; thus, whether serum UA was the cause or consequence of cognitive function in CHF population can not be ascertained in the present design. Second, selecting participants by convenience sampling methods might limit the generalizability of our findings, and probability sampling techniques are recommended for future studies to increase the representativeness of the sample. Additionally, the MoCA is not the gold standard for measuring cognitive function; however, there is increasing evidence that the MoCA is a more comprehensive and suitable tool for screening cognitive impairment in HF patients. Although not designed specifically for the HF population, the MoCA is the only instrument that includes domain subscales and tests all of which most often affected in HF [37]. In addition, the number of CHF patients in the current study was relatively small, and future studies examining the associations between serum UA and cognitive function in CHF patients should employ more samples to increase the validity of our findings. Moreover, some potential confounding variables (e.g., dietary habits [25], systolic blood pressure [69], diuretics, beta-blockers, angiotensin-converting enzyme (ACE) inhibitors, angiotensin II receptor blockers, insulin) were not collected in our study, and future studies should assess more potential variables that may affect the relationship between serum UA and cognitive function in CHF patients. Patients with a history of stroke were excluded, but subtle and silent cerebrovascular disease could not be fully ruled out.

## Conclusions

In conclusion, our cross-sectional study demonstrates that higher levels of serum UA are independently associated with poorer cognitive function in CHF patients after controlling for demographic, medical, and psychological characteristics. Furthermore, elevated serum UA is independently associated with poorer performance on cognitive function in male CHF patients. These findings provide evidence to reevaluate the clinical strategies of reducing serum UA in CHF populations, especially in male CHF populations. Longitudinal studies are strongly warranted to explore whether elevated serum UA increases the risk of cognitive decline in CHF populations and stratified by sex.

## Data Availability

All data analysed during this study are included in this published article and its supplementary information files.

## Abbreviations

CHF: Chronic heart failure
UA: Uric acid
HFrEF: HF with reduced ejection fraction
HFpEF: HF with preserved ejection fraction
NYHA: New York heart association
MoCA: Montreal cognitive assessment
SDS: Zung self-rating depression scale
LVEF: Left ventricular ejection fraction
BMI: Body mass index
STROBE: Strengthening the reporting of observational studies in epidemiology
hs-CRP: C-reactive protein
